# Tuning cancer fate: the unremitting role of host immunity

**DOI:** 10.1098/rsob.170006

**Published:** 2017-04-12

**Authors:** B. Calì, B. Molon, A. Viola

**Affiliations:** 1 Department of Biomedical Sciences, University of Padua, Padua, Italy; 2 Venetian Institute of Molecular Medicine, Padua, Italy

**Keywords:** cancer, immune cells, immunotherapy

## Abstract

Host immunity plays a central and complex role in dictating tumour progression. Solid tumours are commonly infiltrated by a large number of immune cells that dynamically interact with the surrounding microenvironment. At first, innate and adaptive immune cells successfully cooperate to eradicate microcolonies of transformed cells. Concomitantly, surviving tumour clones start to proliferate and harness immune responses by specifically hijacking anti-tumour effector mechanisms and fostering the accumulation of immunosuppressive immune cell subsets at the tumour site. This pliable interplay between immune and malignant cells is a relentless process that has been concisely organized in three different phases: elimination, equilibrium and escape. In this review, we aim to depict the distinct immune cell subsets and immune-mediated responses characterizing the tumour landscape throughout the three interconnected phases. Importantly, the identification of key immune players and molecules involved in the dynamic crosstalk between tumour and immune system has been crucial for the introduction of reliable prognostic factors and effective therapeutic protocols against cancers.

## 1. Immunity and cancer: from immunosurveillance to immunoediting

The contribution of the immune system in shaping tumour outcome has been accepted since the late 1950s, when Lewis Thomas and Frank Macfarlane Burnet proposed the concept of cancer ‘immune surveillance’ [1–3]. According to this model, the immune system is able to limit tumour growth by recognizing antigens expressed on cancer cell precursors and killing them before they become clinically evident, in a process similar to homograft rejection. Indeed, the high probability of genetic mutations in somatic cells of large long-lived animals may be responsible for the genetic alterations characterizing the precursors of malignant cells; consequently, the existence of a mechanism to eliminate these dangerous mutants should be considered an evolutionary necessity for survival [1]. Despite the paucity of data demonstrating efficient immunological eradication of premalignant lesions *in vivo*, overwhelming evidence supports the cancer immune surveillance hypothesis [4]. Severe primary immunodeficiencies in humans, as common variable immunodeficiency, are associated with increased incidence of lymphomas as well as stomach, breast, bladder and cervical cancers [5–8]. Similarly, immunodeficient HIV-infected patients show elevated incidence of tumours associated with oncogenic viruses, such as the HHV8-related Kaposi sarcoma, EBV-related Hodgkin's and non-Hodgkin's lymphoma, HPV-associated cervical cancer and HBV/HCV-related hepatocarcinoma [4]. Notably, although most of the tumours raised in HIV patients seem to be a secondary event of reduced antiviral immunity, CD4^+^ T-cell counts in peripheral blood of HIV-infected individuals inversely correlate with increased cancer risk also for tumours unrelated to viral infections, thus supporting the association between tumour onset and immunosuppression. Moreover, several large cohort studies reported that patients subjected to chronic immunosuppressive therapies to prevent transplanted organ rejection show higher risk for lymphomas as well as other epithelial cancers affecting colon, larynx, bladder, prostate and testis [9]. Several studies in animal models clearly support the concept of cancer immune surveillance. RAG2^−/−^ mice lacking both T and B cells are more susceptible to spontaneous and carcinogen-induced tumours [10]. Moreover, immunocompetent mice are able to rapidly reject cancer cells expressing ligands that can trigger natural-killer (NK) cells or cytotoxic lymphocytes [11,12].

To support the importance of T-cell immunity in controlling tumour progression, it must be anticipated that one of the well-characterized mechanisms of cancer immune evasion is to hijack CD8^+^ T-cell responses basically through the down-regulation of major histocompatibility complex (MHC) class I and the interference with antigen processing pathway in tumour cells [13]. The majority of solid tumours are infiltrated by a large variety of immune cells including CD3^+^ T cells (both CD4^+^ helper and CD8^+^ cytotoxic T cells) and NKp46^+^ NK cells [11,12,14]. Although T and NK cells recruited to the tumour generally show inefficient anti-tumour activity because of the hostile microenvironment created by malignant cells (see below), the quality and quantity of immune infiltrate at the tumour site has been accepted as a prognostic marker of disease progression. In line with this, a revolutionary achievement in the field was the definition of cancer ‘immunoscore’. In 2006, Galon *et al.* [14] provided evidence that the immune contexture in human colorectal cancers acts as a solid predictor of patient clinical outcome. More precisely, the authors discovered that lower incidence of tumour recurrence correlates with intratumoural infiltration of T cells polarized towards a cytotoxic immune response [14]. Nowadays, these observations have been extended to a large variety of human cancers thus appointing the intratumoural infiltration of T lymphocytes as a reliable prognostic indicator for patient outcome [15].

**Figure 1.**
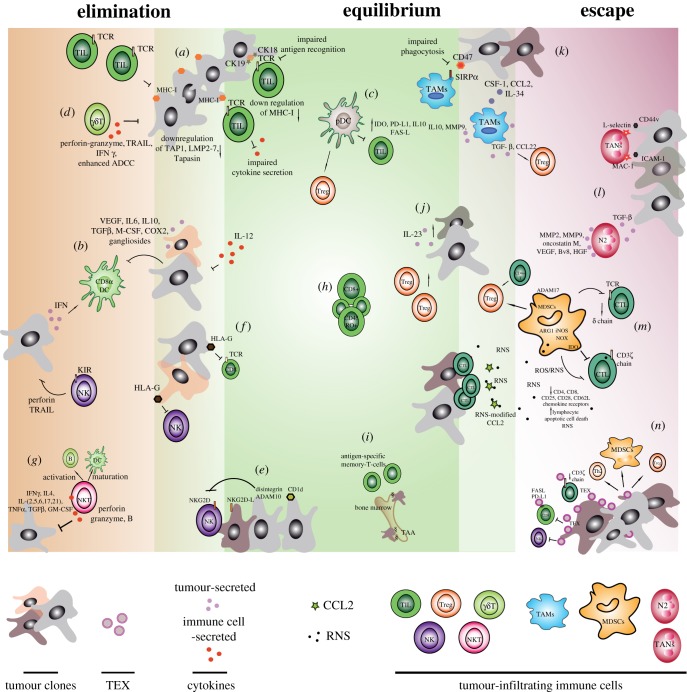
Immune cells contributing to the tumour editing. At the tumour site, the dynamic interplay between immune and malignant cells is characterized by three different phases: elimination (left), equilibrium (middle) and escape (right) [16]. Immune players dictating the three interconnected phases are indicated with lower case labels in the figure and throughout the text.

Although these facts strongly suggest a positive role of the immune response in controlling tumour progression, by killing specific cancer cells and shaping the tumour microenvironment, the immune system has a complex impact on cancer development. Initially developed by Dunn *et al.* [16], the theory of ‘immunoediting’ emphasizes the dual role of the immune system in tumour progression, defining the interaction between immune and malignant cells as a very fine dynamic interplay, characterized by three different phases: *elimination, equilibrium* and *escape*. During the elimination phase, immune cells recruited to the tumour try to mount an efficient anti-tumour immune response to eradicate microcolonies of malignant cells; then, in the so-called equilibrium phase, a fragile dynamic balance between tumour containment (through killing of specific cancer cells expressing antigens or lacking mechanisms to inhibit cytotoxic responses) and selective immune pressure (survival of cancer cells that are resistant to immune responses) is established; finally, during the escape phase, cancer cells take advantage of their key feature of genetic instability to overcome the immune pressure, and the selected tumour clones are able to elude immune response and successfully progress even in an immunocompetent environment [17]. Importantly, throughout the three intertwined phases, different immune cell subsets from both the innate and adaptive immune compartments reach the tumour microenvironment, displaying opposite functions as cancer progresses (figure 1) [18].

## 2. Different immune cell subsets dictate the immunoediting process

### 2.1. Immune cells in the elimination phase

First evidence for the elimination phase of cancer immunoediting in humans derives from the identification of clonally expanded T cells in patients with spontaneously regressed melanoma [19,20]. However, although T-cell infiltration has been observed in many human tumours and has been shown to positively correlate with good prognosis in patients with melanoma, breast, ovarian and colorectal cancer [14,21], their anti-tumour efficacy is very limited. It has indeed been demonstrated that tumour-infiltrating CD8^+^ T cells have impaired cytokine production and proliferation when isolated and cultured *ex vivo*; however, these detrimental features are easily and reversibly abrogated upon restimulation *in vitro* [22,23]. Moreover, developing tumours generally show a downregulation of the MHC class I expression at the cell surface, thus affecting the ability of CD8^+^ cytotoxic T lymphocytes to recognize the malignant cells [24]. Notably, the expression of specific cytokeratins, such as CK18 and its heterodimeric partner CK19, in metastatic carcinoma cell lines has been reported to inhibit interactions between the T-cell receptor (TCR) on CD8^+^ T cells and MHC I by masking the contact motif region [25] (figure 1*a*). Additionally, downregulation or loss of human leucocyte antigen HLA class I molecules have been reported for several epithelial cell cancers and melanoma [26] and it has been associated with an increased propensity for regional lymph-node metastasis [27]. Similarly, it has been documented that during the development of colorectal carcinoma, malignant cells progressively downregulate many proteins involved in antigen processing and presentation, as the transporter associated with antigen processing (TAP1), low-molecular-mass proteins 2 and 7 (LMP2, LMP7) and tapasin [28] (figure 1*a*). Nevertheless, developing cancers secrete type I interferon (IFN) that may promote the accumulation of efficient dendritic cells (DCs) within the tumour microenvironment. Specifically, it has been demonstrated that the conditional deletion of genes involved in type I IFN receptor (IFNR) signalling in the CD8α^+^ DC compartment significantly affects CD8^+^ T-cell priming and tumour rejection [29–31], thus ascribing to CD8α^+^ DCs a crucial role in anti-tumour immunity (figure 1*b*). Besides type I IFN, tumour lesions also produce high amounts of vascular endothelial growth factor (VEGF), interleukin-6 (IL-6), interleukin-10 (IL-10), transforming growth factor-β (TGF-β), macrophage colony-stimulating factor (M-CSF), cyclooxygenase-2 (COX-2) and gangliosides, which can impair the differentiation, maturation and function of DCs [32] (figure 1*b*). Apart from functional DC subsets such as CD8α^+^ DCs, within the tumour microenviroment dysfunctional or tolerogenic DCs such as plasmacytoid DCs (pDCs) have been observed. pDCs collected from tumours show high expression of immunosuppressive molecules such as the indoleamine-2,3-dioxygenase (IDO) enzyme, programmed death-ligand 1 (PD-L1) [33], IL-10 and FAS ligand [34–36], by which pDCs can induce apoptosis or anergy of activated T cells or their conversion in regulatory T cells (Tregs) (figure 1*c*) [37,38].

In addition to the most frequent αβ T cells, T cells expressing γδ TCR (γδ T cells) have been implicated in anti-tumour immune surveillance [39]. Representing only the 5% of total CD3^+^ cells in peripheral blood, γδ T cells do not generally express CD4 or CD8 markers and importantly do not require MHC antigen presentation [40]. They produce a variety of chemokines and cytokines, such as perforin–granzyme, tumour necrosis factor (TNF) and tumour necrosis factor (TNF)-related apoptosis-inducing ligand (TRAIL) and IFN-γ [41–43], by which they are able to inhibit tumour growth and block angiogenesis. Additionally, γδ T cells have been reported to induce tumour killing of FAS- and TRAIL-receptor-sensitive cancers [44–46] to enhance the antibody-dependent cellular cytotoxicity (ADCC) (figure 1*d*) [44–48].

In the absence of MHC class I molecules, an efficient anti-tumour response can be exerted by NK cells, innate lymphocytes that preferentially kill virus-infected cells as well as cells lacking MHC class I antigen expression. NK cell anti-tumour activity is mediated by either a perforin-dependent cytotoxicity [49] or by the secretion of TRAIL, triggering tumour cell lysis [50]. All NK cells and some T cells express on the cell surface the NK group 2 member D (NKG2D) activating receptor which recognizes molecules present on transformed cells [51]. NKG2D ligands, including MHC class I polypeptide-related sequence A (MICA), MHC class I polypeptide-related sequence B (MICB) and six different UL16-binding proteins (ULBP) in humans [52], and the UL16-binding protein MULT1, RAE-1 and H60 proteins in mice [53], have been reported to be upregulated in normal cells after exposure to ionizing radiation and UV light, thus suggesting a crucial role in alerting the immune system of the presence of potentially dangerous cells [54]. These findings have been also supported by *in vivo* studies showing that the injection of cancer cells transfected with the NKGD2 ligands RAE-1 and H60 results in a rapid rejection of the tumour by NK and CD8^+^ T cells [11,55]. This notwithstanding, downregulation of MICA/MICB has been observed in stem-like breast cancer cells, due to the altered expression of the oncogenic microRNA miR20a [56]. Importantly, in hypoxic conditions typical of tumour lesions, cancer cells upregulate the expression of disintegrin and metalloproteinase containing-domain 10 (ADAM10), which has been reported to cleave MICA/MICB from cell surface of prostate and breast cancer cell lines, thus contributing to impair NK cell-mediated tumour cell elimination (figure 1*e*) [57].

Among the mechanisms to avoid NK cell-mediated killing, cancer cells can integrate MHC class I containing vesicles released from platelets into their own cell membrane [58]. Further, tumour cells may express the non-canonical MHC molecule HLA-G. HLA-G is expressed in human trophoblasts and plays a crucial role in protecting the placenta from rejection [59]. HLA-G negatively affects immune cell functions by binding different receptors on different cell subsets, including the killer-cell immunoglobulin-like receptors KIR, expressed on NK cells, and CD8, expressed on cytotoxic T cells [60–62]. Not surprisingly, HLA-G expression, which has been observed in melanoma, glioma, breast, lung and ovarian cancer, is associated with poor patient survival [60–67] and plasma levels of soluble HLA-G, secreted by tumour cells as a result of alternative splicing, have been shown to correlate with the presence of circulating tumour cells and disease progression (figure 1*f*) [68].

Lastly, a relevant role in tumour immune surveillance has been documented also for NKT cells (NKT) [69]. NKT cells express a TCR but, differently from conventional T cells, which require antigen presentation by MHC molecules, they recognize lipid antigens presented by the non-polymorphic MHC class I-like CD1d molecules [70,71] and show cytotoxic activity. Thus, sharing features with both NK cells and T cells, they represent the bridge between the innate and adaptive immunity. NKT cells have been intriguingly reported to inhibit tumour growth in several murine studies, including a p53-deficiency model and a TRAMP model [72,73]. Notably, once activated, they are able to hinder cancer growth either directly or indirectly. Indeed, NKT cells are able to directly kill tumour cells by an NK-like effector mechanism, based on perforin and granzyme B cytotoxicity [74,75]. Additionally, by releasing a huge plethora of cytokines, including primarily IFNγ and IL4, followed by IL2, IL5, IL6, IL10, IL17, IL21, TNFα, TGFβ and GM-CSF [76], NKT cells are indirectly involved in inhibiting tumour angiogenesis [77] and enhancing antigen-specific immune responses, by the induction of dendritic cell maturation and B-cell activation (figure 1*g*) [78]. The reduction of circulating NKT cells has been proposed as an independent predictor of poor overall survival and disease-free survival in patients with head and neck squamous cell carcinoma [79]; similarly, high NKT cell infiltration of primary colorectal carcinomas has been considered an independent prognostic factor for both overall survival and disease-free survival [80].

Nonetheless, although CD1d expression has been documented in several solid tumours, such as prostate and breast cancers, renal cell carcinoma, malignant glioma and paediatric medulloblastoma [69], a significant reduction of number [81] and function [82] of peripheral blood NKT cells was found in patients with progressive multiple myeloma, prostate cancer and other solid malignancies [69], thus indicating the induction of tumour immune escape.

### 2.2. Immune cells dictating the equilibrium phase

The equilibrium phase or, as properly suggested, the cancer immune equilibrium [83], represents a critical step of cancer immunoediting. At this stage, tumour clones that elude and survive the elimination checkpoint coexist with host immune cells in a dynamic equilibrium. The precise mechanisms dictating the duration and progression of this complex phase remain largely unknown and, for long time, a foremost question in the field was whether or not this phase de facto exists. Until recently, a major limit to address this inquiry was the lack of mouse models to experimentally investigate this condition. In 2007, a seminal study by Koebel *et al.* [84] unequivocally confirmed the occurrence of the equilibrium phase in immunocompetent hosts, highlighting the mechanisms by which the immune system might control cancer growth and coincidently sculpt tumour immunogenicity. Indeed, by using a mouse model of primary chemical carcinogenesis, authors showed that the ablation of specific cellular subsets orchestrating adaptive immunity enables the outgrowth of ‘dormant’ tumour clones, which could be restrained by effective immune responses [84].

Later on, additional investigations in different murine models supported the notion that host immunity represents an effective weapon controlling occult tumours [85]. Nonetheless, former evidence that a competent immune system could maintain tumours in a ‘dormant’ state was provided by clinical observations of transplantation of latent tumour cells in organ donors into immunosuppressed hosts [86] and by pioneering studies on leukaemia–lymphoma cell transplantation in pre-immunized mice [87,88]. At first, the equilibrium phase paralleled the old concept of ‘tumour dormancy’, where quiescent cancer cells silently survive throughout the body for a long period before growing to form full-blown tumours, in a phenomenon defined as cancer relapse [89]. A similar condition is represented by the appearance of the minimal residual disease in both haematopoietic and solid tumours. It has been documented that circulating, disseminated tumour cells still survive in cancer patients who are free of disease recurrence for more than 20 years [90]. Nonetheless, the equilibrium state goes beyond the traditional concept of tumour dormancy as it always refers to an undefined but long-lasting phase in which host immunity relentlessly blocks the outgrowth of latent tumour clones. Different scenarios characterize this process; indeed, on the one hand, it is possible that rare tumour cells remain completely quiescent for several years, being constantly eliminated by the immune system; on the other, the durable interplay between host immune cells and proliferating tumour clones ultimately establishes a selective pressure that sculpts tumour immunogenicity. Only tumour cells that become phenotypically irrelevant for adaptive immunity can successfully evade the immune surveillance, progressively originating visible tumours [91].

As mentioned before, several events occurring in the equilibrium phase still remain mechanistically unresolved and temporally undefined; even more importantly, a burning issue to be addressed is how and when the balance between tumour cell growth and immune cell control gets broken. To delve into the question, we need first to analyse which are the molecular and cellular determinants that uphold cancer immune equilibrium.

According to Dunn and colleagues, host immunity differentially participates to cancer immunoediting, with both innate and adaptive immunity playing crucial roles in the elimination and escape phases, but only the adaptive immune cell subsets involved in maintaining the equilibrium framework [16,84]. Tumour-infiltrating lymphocytes (TILs) play a pivotal role in controlling cancer cell growth by actively promoting tumour dormancy [92]. As previously mentioned, high numbers of CD8^+^ cytotoxic T lymphocytes and CD45RO^+^ memory T cells within the primary tumour lesion remarkably correlated with positive clinical outcome in different cancers [93]. In addition to the number and type of tumour-infiltrating immune cells, an important factor conditioning the control of tumour growth is their relative positioning within the neoplastic mass. Indeed, usually TILs efficiently infiltrating the tumour core display productive anti-tumoural responses, thus sustaining the elimination phase; on the other side, lymphocytes that are trapped at the periphery do not always exert anti-tumoural activity (figure 1*h*) [93,94].

By analysing tumour relapse in 18 acute myeloid leukaemia paediatric patients in remission, Montagna *et al.* [95] documented the presence of anti-tumour cytotoxic T cells (CTL) in the peripheral blood of children who did not experience tumour relapse, whereas relapsing patients did not present circulating anti-tumour CTL. Indeed, T lymphocytes play a leading role in the achievement and maintenance of this ‘equilibrium’ condition. Interestingly, the bone marrow represents an elective site for quiescent tumour cells and for the maintenance of anti-tumour memory T cells [96]. By using an athymic mouse model, challenged with Gal-expressing tumour cells and subsequently transferred with Gal-specific CD8^+^ T cells, it has been shown that the bone marrow acts as an active reservoir of tumour-associated antigens, derived from dormant tumour cells (figure 1*i*). Tumour antigens at this site promote the survival of tumour antigen-specific memory T cells over irrelevant memory T cells [96]. Clinical observations further confirmed this evidence: breast cancer patients having cells positive for the tumour marker cytokeratin in the bone marrow showed a higher proportion of CD4^+^ and CD8^+^ memory T cells compared with healthy subjects [97].

TILs comprise distinct cell subsets, including CTLs, T helper 1 (Th1), T helper 2 (Th2), T helper 17 (Th17), Treg, γδT cells, NK and NKT cells, that actively mould the tumour microenvironment. Nonetheless, although their role has been exhaustively investigated in both the elimination and the escape phases, their specific contribution to the establishment and maintenance of cancer equilibrium is not clear. A recent report [98] investigated this issue by analysing the percentage of different immune cell populations among TILs in either dormant or progressive sarcoma in a mouse model. Data from this study suggested that the intratumoural and splenic accumulation of CTL, NK and γδT cells, with a low percentage of NKT cells and a normal/low percentage of splenic and intratumoural Treg cells may represent robust prognostic markers for immune-mediated dormancy in mouse sarcomas [98].

Cytokines play an important role in regulating cancer and immune cell dynamics in the equilibrium phase. An elegant study from Teng *et al.* [99] showed that in neoplastic lesions, induced by low-dose regimen of methylcholanthrene, a prolonged equilibrium state could be maintained by the concurrent activity of the cytokines IL-23 and IL-12, with the former promoting cancer survival and the latter inhibiting cancer outgrowth (figure 1*j*).

Clinically, the equilibrium phase represents an attractive target to be investigated: it is in this temporal window that tumour cells undergo fine editing processes based on both genetic and epigenetic changes, ultimately making them immuno-silent clones. On the other hand, at this stage the host immune system still preserves the ability to stem cancer cell outgrowth. The identification of the distinct elements promoting or breaking this equilibrium may pave the way for the definition of novel targetable checkpoints.

### 2.3. Immune cells responsible for the tumour escape

Since growing tumours represent persistently damaged tissues, a common feature of all cancers is the establishment of a chronic inflammatory microenvironment, consisting of a multitude of chemical signals and different corrupted resident or purposely recruited immune cells crowding the tumour to heal the injury [100]. Smouldering inflammation has been so far associated with increased risk for many types of cancer, including bladder, cervical, gastric, intestinal, oesophageal, ovarian, prostate and thyroid cancer [101]. Although the different number, type and location of tumour-infiltrating immune cells reflect peculiar biological aspects of individual cancers, the most represented immune cells accumulated at the tumour mass share a myeloid origin, and include macrophages, granulocytes and myeloid-derived suppressor cells (MDSCs), which are generated from bone marrow as a consequence of a cancer-induced abnormal myelopoieis [102,103].

Macrophages flocked to the tumour, the so-called tumour-associated macrophages (TAMs), are generally recruited by the activation of CSF1R by CSF-1 or IL-34, as well as by the chemokine CCL2 [104]. Macrophages have been shown to be able to eliminate malignant cells exploiting multiple killing mechanisms. Indeed, macrophages can release apoptosis-inducing soluble factors, such as nitric oxide (NO) and TNFα [104–107], exert phagocytosis based on the expression of signal molecules on the surface of tumours cells, such as phosphatidylserine and calreticulin, and clear viable antibody-coated tumour cells [108]. However, tumour cells escape macrophage killing through the upregulation of the ‘don't eat me’ signal CD47 [109], which is a ‘self’ integrin-associated protein expressed by healthy cells which binds to the signal regulatory protein α (SIRPα) on macrophage cell surfaces to inhibit phagocytosis (figure 1*k*) [110].

According to their activation state, macrophages have been historically classified in two functionally polarized states: classic M1 or alternative M2 macrophages [111]. IFN-γ represents the prevailing cytokine associated with M1 cells, which are commonly induced during bacterial or viral infections to promote the inflammatory responses against pathogens. On the other side, the M2 subset includes macrophages that have been exposed to IL-4, IL-10, IL-13 and glucocorticoid hormones, have an anti-inflammatory phenotype, and participate in tissue regeneration [111]. Within the tumour microenvironment, M1 macrophages express high levels of IL-12 and low levels of IL-10 and are tumouricidal; on the other hand, M2 macrophages secrete high levels of IL-10 and low levels of IL-12, display poor antigen presenting capacity, and promote angiogenesis, tumour invasion and metastasis [112–114]. TAMs generally resemble functional features typical of the M2 anti-inflammatory phenotype; nonetheless, they can also secrete pro-inflammatory cytokines, such as IL-6, that are involved in oncogenic programmes during tumour development [115]. It is important to note, however, that the M1/M2 classification is a schematic under-representation of a complex functional spectrum acquired by macrophages in response to environmental as well as tumour-derived stimuli [116]. Thus, TAMs represent a cell population characterized by plasticity and capable of rapidly shaping its phenotype and activity in response to tumour environmental cues. Indeed, during tumour progression, TAMs functionally switch from an M1-polarized state, which initially orchestrates the immune responses against cancer cells, to an M2-like phenotype, which oppositely sustains tumour progression [115].

TAMs suppress anti-tumoural adaptive immunity acting on the intratumoural IL-10/IL-12 balance, critical for priming T-lymphocyte responses [117,118], and secreting an array of cytokines and chemokines involved in development and recruitment of immunosuppressive immune cells, such as Tregs, immature DCs and MDSCs (see below) [119].

Similarly to macrophages, tumour-associated neutrophils (TANs) have been shown to shift from an anti-tumoural N1 phenotype to a pro-tumoural N2 phenotype after TGF-β exposure, which is frequently secreted by cancer cells [120]. Several reports have shown that TANs secrete MMP2, MMP9, oncostatin M, VEGF, Bv8 and hepatocyte growth factor, thus sustaining extracellular matrix remodelling, tumour invasion and angiogenesis [121–123]. Importantly, the presence of neutrophils has been reported to correlate with reduced survival in head and neck and breast cancer patients [124]. In mouse models of cancer, neutrophils have been shown to play a critical role in driving tumour cell dissemination and metastasis, acting as a bridge to facilitate interactions between cancer cells and metastatic sites; this process seems to be driven by ICAM-1 and CD44v, expressed on cancer cells and their molecular partners MAC-1 and L-selectin, respectively, expressed by neutrophils [17]. Additionally, several studies reported that neutrophils can exploit the NETosis process, by which they generally entrap microbes during sepsis [125], to sustain cancer cell dissemination and metastasis, facilitating the adherence of cancer cells to blood vessels and their extravasation into target organs (figure 1*l*) [17,126,127].

The accumulation of MDSCs within the tumour microenvironment is a process common to both mouse and human tumour development [128]. MDSCs are generally defined as a heterogenous population of myeloid cells, including different subsets of myeloid cells at different stages of maturation, with an extraordinary ability to suppress T-cell responses [129]. Although sharing some features with monocytes (M-MDSCs) or neutrophils (PMN-MDSCs) [102,130], MDSCs differ from mature myeloid cells in terms of phenotype [131] and activity (reviewed by Kumar *et al.* [128]). Differently from TAMs and TANs, which may exert a dual role in cancer progression, the key feature of MDSCs relies on their immunosuppressive capacity, and therefore these cells play a negative role in the fight between the immune system and tumours.

Expansion of an immunosuppressive MDSC population is frequently observed in human cancers as well as in many other pathological conditions and is the result of several different factors (reviewed in [132]). Cytokines, prostaglandins and other factors produced mainly by tumour cells stimulate myelopoiesis and, at the same time, inhibit the differentiation of mature myeloid cells, thus promoting the expansion of MDSC. The second group of factors is produced mainly by activated T cells and tumour stroma, and is involved in directly activating MDSCs [132]. MDSCs generated in the bone marrow migrate into the tumour and peripheral lymphoid organs, mainly attracted by the chemokines CCL2 and CCL5, and receive stimuli that activate their immunosuppressive properties (figure 1*m*) [133].

The immunosuppressive properties of MDSCs involve multiple mechanisms. First, MDSCs induce the activation and expansion of regulatory T-cell (Treg) population, promoting antigen-specific natural Treg clonal expansion and naive CD4^+^ T-cell conversion into induced Tregs by a mechanism that requires tumour-associated antigen capture, processing and presentation by MDSCs themselves [134]. Additionally, it has been reported that intratumoural MDSCs directly mediate the CCR5-dependent recruitment of Tregs by the secretion of CCL3, CCL4 and CCL5 chemokines [135]. The second immunosuppressive mechanism is based on depletion of nutrients required by lymphocytes (i.e. cysteine, arginine and tryptophan [136] depletion through arginase-1 (ARG1)-dependent consumption), which causes TCR δ-chain downregulation and the proliferative arrest of antigen-activated T cells [137]. Moreover, MDSCs interfere with lymphocyte trafficking and viability by expressing at the plasma membrane ADAM17, which cleaves key proteins for T-cell recirculation to lymph nodes, and galectin-9 (GAL-9), which induce T-cell apoptosis. Finally, MDSCs produce reactive oxygen species (ROS), nitric oxide (NO) and other reactive nitrogen species (RNS) by the synergistic activity of ARG1, iNOS and NADPH oxydase enzymes [102]. While arginase hydrolyses arginine into ornithine and urea, iNOS oxidizes arginine to citrulline and produces NO. Importantly, NO produced by iNOS may rapidly react with ROS within the tumour lesion and thus produce RNS, such as peroxynitrite, that are very toxic for many cells, and in particular for T cells. Peroxynitrite induces apoptotic cell death in lymphocytes by interfering with protein tyrosine phosphorylation via nitration of tyrosine residues [138] or by nitrating the voltage-dependent anion channel, a component of the mitochondrial permeability transition pore [139]. RNS affect T-cell recognition of tumour antigens in several ways: by interfering with antigen processing and presentation or by inhibiting T-cell activation [140]. Indeed, persistent exposure to RNS modulates the expression and signalling properties of several T-cell proteins, including the CD3ζ chain of the TCR complex [138,141,142]. Intriguingly, RNS can induce post-translational modifications, such as nitration and nitrosylation, of chemokines and chemokine receptors, thus blocking T-cell recruitment, promoting local immune dysfunction and preventing effective response (figure 1*m*) [94].

In addition to these mechanisms that require the direct action of immunosuppressive immune cells, cancer cells can directly target anti-tumour immune response by the release of immunosoppressive exosomes. Exosomes are small (30–150 nm) extracellular vesicles shuttling proteins and nucleic acid, mainly mRNAs and miRNAs [143]. Tumour exosomes (TEXs) have been identified in tumour tissues and in the serum of cancer patients, and have been shown to affect tumour immunity [144]. Although TEXs do not appear to be rapidly internalized by T cells, they can deploy several interfering mechanisms to either directly or indirectly hamper anti-tumoural immune response. TEX-driven direct suppression of immune cells is generally the result of an apoptotic process, triggered by FASL and PD-L1 [145,146]. Moreover, it has been demonstrated that TEXs affect CD3ζ chain expression and push T cells towards a Th2 phenotype, by triggering NF-κB and STAT3 activation [143]. TEXs can also inhibit NK cell cytotoxicity, impair monocyte differentiation, favour MDSC differentiation and enhance Treg induction within the tumour microenvironment [147–149]. This is also mediated by transfer of miRNAs such as miR-21, miR146-a, miR-155 and miR-568 that affect the differentiation and functions of immune cells (figure 1*n*) [143].

## 3. The era of cancer immunotherapy

The deeper understanding of the crucial role of immune system in either blocking or sustaining tumour progression has prompted the development of various immunotherapeutic approaches against cancer. Basically, the concept of cancer immunotherapy is to boost host immunity against cancer cells and, at the same time, to block corrupted immune elements responsible for promotion of tumorigenesis. Over the last few decades, several immunotherapeutic strategies have been proposed and tested in both preclinical animal models and clinical settings [150]. Among them, the most successful protocols appeared to be cancer vaccination, adoptive cell therapy (ACT) and the targeting of immune checkpoints.

Cancer vaccines are mainly based on the stimulation of anti-tumour immune responses induced by tumour antigens. Several clinical trials have been successfully conducted for the treatment of premalignant lesions or for the prevention of recurrences after treatment of the primary malignancies [151,152]. One the most promising protocols for the treatment of established cancers consists in the administration of Sipuleucel-T, a cancer vaccine based on pulsing of autologous APC with a chimeric protein composed of phosphatase acid prostatic protein fused to GM-CSF, which has been successful in metastatic, castration-resistant prostate cancer patients [153]. However, a major drawback in the vaccination approach relies on the persistence of immune cell suppression despite the exogenous immunization. Therefore, combinatorial strategies consisting of vaccination supported by other immune boosting approaches, such as ACT and immune checkpoints blockade, have been developed for cancer treatment [154–158].

Specifically, ACT is based on the *ex vivo* isolation and IL-2 sustained expansion of antigen-specific T cells for adoptive transfer back to patients [159–161]. One common strategy of ACT presupposes the isolation of autologous T cells infiltrating the tumour, in order to activate them *ex vivo* and reinfuse them into the patients [162]. A more efficient ACT strategy exploits the opportunity of genetically engineering autologous T cells with lentiviral or retroviral tools to express specific tumour-associated antigen-recognizing TCR [160]. However, although clinical success has been reported for haematologic malignancies and melanoma, the efficacy of ACT in the treatment of solid tumours has been very limited so far, because of the very low persistence and functionality of engineered T cells *in vivo* [163]. The expression of chimeric antigen receptors (CARs), constituted by an antigen-binding domain, usually a single-chain variable fragment (scFv), fused with an intracellular T-cell signalling domain such as CD3-ζ and one or two co-stimulatory domains, represents another very promising immunotherapeutic approach [160]. A very recent report [164] showed objective regression of lung metastasis after the infusion of CD8^+^ cells, isolated from biopsies of a metastatic colorectal patients and targeting a specific G12D KRAS mutation. Notably, the treatment with CD8^+^ cells targeting mutant KRAS has been successful against a cancer that expressed both the specific G12D mutation and the HLA-C*08:02 restriction molecule, thus providing key evidence of the importance of HLA molecule downregulation for tumour immune evasion [164]. An important issue to be considered for cancer immunotherapy is that transferred T cells do not efficiently reach the tumour core because of the hostile, immunosuppressive microenvironment and that administration of molecules interfering with the mechanisms responsible for tumour-induced immunosuppression, such as RNS production or CSF-1 signalling, may improve the efficacy of ACT [94,165].

The need for alternative strategies aimed at interfering with the negative regulators of anti-tumour immunity prompted the development of immune checkpoint blocking agents, such as monoclonal antibodies against key immunosuppressive molecules including cytotoxic T-lymphocyte antigen 4 (CTLA-4) and programmed cell death protein-1 (PD-1). Specifically, the first immune checkpoint receptor to be clinically targeted was CTLA-4, a type 1 transmembrane glycoprotein mainly expressed on activated T cells and commonly upregulated to inhibit T-cell function, by competing for the binding of CD28 with B7 molecules [166,167]. Importantly, the two anti-CTLA-4 antibodies ipilimumab and tremelimumab showed efficient and persistent clinical response in several patients with advanced melanoma and non-small-cell lung cancer [168]. Also, efficient antibodies anti-PD-1 and PDL-1 have been developed and tested in several malignancies [169]. The primary role of PD-1 is to inhibit effector T-cell activity and enhance the function and development of Tregs [170–172], by binding the PD-L1 and PD-L2 ligands, expressed on immune cells and several types of solid tumours [173–175]. Different anti-PD-1 antibodies, such as nivolumab and pembrolizumab, have demonstrated efficacy in patients with advanced melanoma, non-small-cell lung cancer, renal cell carcinoma and other solid tumours, and have been approved for the treatment of advanced, metastatic melanoma, lung cancer, metastatic renal cancer and Hodgkin’s lymphoma [169]. Importantly, while CTLA-4 seems to regulate early T-cell activation, PD-1 inhibits effector T-cell activity in the effector phase within tissue and tumours. Nevertheless, despite documented clinical success of checkpoint inhibitor blockade, more than 70% of cancer patients remain resistant to these treatments. Importantly, recent studies have demonstrated a crucial role for the gut microbiota in promoting the efficacy of anti-cancer therapy, pinpointing clinical strategies that may benefit from modulating the microbiota composition, such as cyclophosphamide, platinum salts, as well as immune checkpoint inhibitor administration [176]. Indeed, the efficacy of cyclophosphamide is partly due to intestinal bacteria, against which the host becomes immunized during the treatment, accumulating anti-commensal effector pTh17 and memory Th1 CD4^+^ T cells, which are necessary for the anti-cancer effect [177,178]. Further, the anti-tumour efficacy of oxaliplatin has been demonstrated to depend upon the priming of the myeloid cells by the gut microbiota for the release of ROS that contribute to genotoxicity and tumour reduction [179]. Finally, the success of CTLA-4 blockade has been shown to be facilitated by constituents of the microbiota, especially certain *Bacteroides* spp. and Burkholderiales, which control tumour progression by stimulating Th1 immune responses during anti-CTLA-4 therapy [180,181].

Therefore, the development of novel anti-tumour combinatorial strategies that also promote the manipulation of the microbiome towards a status that promotes immune-mediated tumour control remains an urgent need for biomedical researchers.

## 4. Conclusion

So far, evidence has shown that solid cancers are largely infiltrated by immune cells capable of shaping tumour progression [11,12,14]. Initially accepted in the late 1950s, the theory of immune surveillance suggested a positive role for the immune response in controlling tumour progression by killing specific cancer cells [1]. However, the findings of different immune cell subsets with opposite functions within the tumour microenvironment supported the more recent theory of immunoediting, which emphasizes the dual role of the immune system in tumour progression. According to this model, the interaction between immune and tumour cells is a very fine dynamic interplay, characterized by three different intertwined phases: elimination, equilibrium and escape [16]. During the elimination phase, conventional T cells [14,21,93], γδ T cells [39], NK [11,12] and NKT cells [69] try to mount efficient anti-tumour immune responses to kill malignant cells; however, they have to face continuously changeable tumour clones able to downregulate tumour antigens and MHC class I molecules. Then, in the equilibrium phase, a fragile balance between tumour containment and selective immune pressure by cytotoxic T cells and antigen-specific memory T cells is dynamically maintained thanks to a peculiar cytokine milieu [93,99,182]. Finally, during the escape phase, cancer cells overcome the immune pressure and elude immune response to successfully progress and eventually disseminate throughout the body. This phase is characterized by the involvement of immune cells of myeloid origin, such as TAMs [112], neutrophils [120,124] and MDSCs [128,130] that collectively suppress T-cell activity, sustain tissue remodelling and favour cancer progression and dissemination [102]. Importantly, the identification of key immune players and molecules involved in the dynamic crosstalk among tumour and immune system has been crucial for the introduction of reliable prognostic factors, such as the immunoscore [14] and effective therapeutic protocols for the treatment of specific tumours [153,164,168]. However, deeper investigations of the signalling pathways regulating the three immunoediting phases, and particularly the equilibrium one, are still fundamental for the development of other broader effective therapeutic strategies able to boost the immune system against most cancers.
